# WITHDRAWN: Inhibition of MicroRNA miR-92a Inhibits Cell Proliferation in Human Acute Promyelocytic Leukemia: This article has been withdrawn due to the fact that it is published in three different journals.

**DOI:** 10.4274/Tjh.2012.0171

**Published:** 2013-06-05

**Authors:** Mohammadreza Sharifi, Rasoul Salehi, Yousof Gheisari, Mohammad Kazemi

**Affiliations:** 1 Mohammadreza Sharifi, Rasoul Salehi, Yousof Gheisari, Mohammad Kazemi Pediatrics Inherited Diseases Research Center & Department Of Genetics And Molecular Biology, School Of Medicine, Isfahan University Of Medical Sciences, Isfhan, Iran

**Keywords:** microRNA, miR-92a, Acute promyelocytic leukemia, Locked Nucleic Acid

## Abstract

**Objective:**

MicroRNAs (miRNAs) are endogenous non-coding RNAs, 19-25 nucleotides in length involved in post-transcriptional regulation of gene expression in a considerable majority of mRNAs. In many tumors, up- or down-regulation of different miRNAs has been reported. In acute myeloid leukemia up-regulation of miR-92a has been reported in humans in vitro studies. In this study it is mainly aimed to assess the effect of inhibition of miR-92a in viability of acute promyelocytic leukemia (APL).

**Materials and Methods:**

We performed inhibition of miR-92a in an acute promyelocytic leukemia (APL) cell line (HL-60) using locked nucleic acid (LNA) antagomir. At different time points after LNA-anti-miR92a transfection, miR-92a quantitation and cell viability were assessed by qRT-real-time-polymerase chain reaction (PCR) and MTT assays. The data was processed using the ANOVA test.

**Results:**

Down-regulation of miR-92a in APL cell line (HL-60) by LNA antagomir extensively decreased cell viability in APL. Cell viability gradually decreased over time as the viability of LNA-anti-miR transfected cells was less than 50% of untreated cells at 72 h post-transfection. The difference of cell viability between LNA-anti-miR and control groups was statistically significant (p<0.024).

**Conclusion:**

Based on our findings, it is concluded that inhibition of miR-92a may represent a potential novel therapeutic approach for treatment of APL.

**Conflict of interest:**

None declared.

## Abstract (tr)

**Amaç:**

MikroRNA (miRNA) lar endojen protein kodlamayan RNA’lar, 19-25 nükleotit uzunlukta olup mRNA’ların önemli bir çoğunluğunda post-transkripsiyonel gen ekspresyon regülasyonunda yer almaktadırlar. Çoğu tümörde, farklı miRNA’ların up- veya down-regülasyonu rapor edilmiştir. Akut myeloid lösemide miR-92a’nın up-regülasyonu insan in vitro çalışmalarında bildirilmiştir. Bu çalışmada esas olarak, akut promyelositik lösemi (APL) de miR-92a inhibisyonunun etkisinin değerlendirilmesi amaçlanmıştır.

**Gereç ve Yöntemler:**

Akut Promyelositik Lösemi (APL) hücre dizisinde (HL-60) locked nükleik asit (LNA) antagomir kullanarak miR-92a’nın inhibisyonunu gerçekleştirdik. LNA-anti-miR92a transfeksiyonu sonrası farklı zamanlarda, miR-92a kantitasyonu ve hücre viabilitesi qRT-Real-Time-polimeraz zincir reaksiyonu (PCR) ve MTT testleri ile değerlendirildi. ANOVA testi kullanılarak veriler işlendi.

**Bulgular:**

APL hücre dizisinde (HL-60) LNA antagomir ile miR-92a down regülasyonu APL’de hücre viabilitesini belirgin ölçüde azalttı. Post-transfeksiyon sonrası 72. saatte, LNA-anti-miR transfekte hücreler muamele edilmemiş hücrelerin %50 sinden azı olduğunda, hücre viabilitesi kademeli olarak zaman içinde azaldı. LNA-anti-miR ve kontrol grupları arasında hücre canlılığı açısından farklılık istatistiksel olarak anlamlıydı (p<0.024).

**Sonuç:**

Bulgularımıza göre, miR-92a inhibisyonunun APL tedavisi için yeni bir potansiyel terapötik yaklaşım olabileceği sonucuna varılmıştır.

## INTRODUCTION

MicroRNAs (miRNAs) are post-transcription regulators of gene expression involved in many cellular biological and pathological activities [1]. Initially discovered in 1993 in Caenorhabditis elegans should be italicized, miRNAs were next revealed in many other organisms, including human beings, and proved to play crucial roles in regulation of gene expression. They are composed of the short ribonucleic acid (RNA) chain of 19-25 nucleotides [2]. MiRNAs are involved in various biological processes, including the cell cycle, differentiation, growth and development, metabolism, aging and apoptosis [3,4,5,6,7]. Deregulation of miRNAs expression, including both down- and up-regulation of their biogenesis, mimics the phenomenon attributed to the oncogenes and tumor suppressor genes, depending on the target mRNA regulated by particular species of miRNAs [8,9,10,11,12]. In addition, many reports favoring their essential function In addition, many reports favor their essential function in a wide range of diseases, including heart and cardiovascular diseases; rheumatological, infectious, inflammatory, autoimmune, metabolic disorders and cancers [8,13,14].

Acute myeloid leukemia (AML) is a disorder of hematopoietic stem cells, accompanied with obstruction in hematopoietic cell differentiation and increased clonal neoplastic proliferation. If remained untreated, this malignancy leads to death within a few weeks to several months [15]. Despite extensive studies, the biological and pathophysiological of AML are not yet clearly known [16]. MiRNAs as influential players in cell function can potentially shed light on the pathogenesis of this disorder and could be considered as a potential novel therapeutic option, especially in cases where conventional therapies are less beneficial [17,18,19].

Recent studies on miRNAs in relation to AML revealed many up- and down-regulation events, some correlated with particular cytogenetic aberrations. It has been shown that there is an increased expression of miR-92a in AML[20,21]. In addition, this miRNA has been shown to negatively regulate p63 in CD32 cells (a murine myeloid cell line) leading to increased proliferation of these cells [22]. MiR-92a is a member of the miR-17-92 cluster located on chromosome 13q31.3. It has been suggested that this cluster may function as the oncogene in some other malignancies, including chronic myelocytic leukemia [23].

Based on this evidence, we have proposed to examine the effects of miR-92a inhibition on HL-60 cell line proliferation and viability as a basic preliminary step in developing a novel therapeutic strategy for AML. According to the American-French-British (FAB) classification, AML is divided into eight subtypes, M0-M7 [15]. HL-60 cells are suitable in vitro model of AML-M3, also known as acute promyelocytic leukemia (APL).

## MATERIALS AND METHODS

### Cell Culture

The HL-60 cell line (Human Promyelocytic Leukemia: APL) was purchased from the National Cell Bank of Iran (NCBI; Pasteur Institute, Iran) with Cytogenetic: 44,X,-X,-5,dic(5;17)(q11;p11)del(7)(p?),der(7)t(5;7)(q11;q?31)t(5;16)(q11;?),add(8)(?),der(9)del(9)(p2?)t(9;14)(q2?;q2?),del(10)(p?),ins(11;8)(q13?;?),der(14)t(14;15)(q1?;q?),15,der(16)(5;16)(q?;q?22~24),der(16)t(7;16)(?;q?22~24),+18. The cell culture was maintained in Roswell Park Memorial Institute (RPMI) 1640 (Gibco, Paisley, UK) supplemented with fetal calf serum (FCS; Gibco, Paisley, UK) 15% v/v, 100 U/mL of penicillin and 100 µg/mL of streptomycin (Gibco, Paisley, UK) in an air-saturated and humid atmosphere consisting of 5% CO2 in 25-cm2 culture flasks (Nunc, Roskilde, Denmark) at 37°C. The cells were passaged twice weekly to maintain an exponential growth phase.

### Cell Transfection

The nucleotide sequences of miR-92a were obtained from www.mirbase.org as UAUUGCACUUGUCCCGGCCUGU (accession number MIMAT0000092). The miRCURY LNA microRNA Inhibitor™ for hsa-miR-92a and microRNA inhibitor negative control (scrambled) oligonucleotides were purchased from Exiqon, Denmark. Both oligonucleotides were labeled at the 5´ end with fluorescent dyes, 6-FAM, for subsequent detection of transfected cells.

HL-60 cell transfection was performed by using the X-treme GENE siRNA Transfection Reagent™ (Roche, Mannheim, Germany) according to the manufacturer instructions. Briefly, 5x105 cells in the exponential growth phase were cultured in six-well culture plates (Nunc, Roskilde, Denmark) containing 1.8 mL RPMI 1640 per well without antibiotics and FCS. The 50 pmol miRCURY LNA microRNA inhibitor™ was mixed with 5 µL X-treme GENE siRNA Transfection Reagent™ in 200 µl Opti-MEM I Medium™ (Gibco, Paisley, UK) and incubated for 15 min at room temperature. The complex was then added to the cells and swirled cautiously to ensure even distribution over the entire plate surface. After 8 h of incubation, the FCS and antibiotics were added, and the cells were incubated for the 24, 48, and 72 h. Untreated cells and cells transfected with scrambled-LNA were cultured parallel to the LNA-anti-miR transfected cells. Efficiency of the transfection was examined by flow cytometry and fluorescent microscopy. LNA was conjugated with 6-FAM™ Fluorescein (6-carboxyfluorescein) in order to detect and quantify LNA transfected cells by fluorescent microscopy and FACSCalibur flow cytometer (BD, USA).

### Reverse Transcriptase microRNA Real Time Polymerase Chain Reaction

Reverse transcriptase (RT) microRNA real time polymerase chain reaction (PCR) was performed to determine the efficiency of miR-92a inhibition by LNA-anti-miR. Briefly, the total cellular RNA was extracted 24, 48, and 72 h post transfection with the miRCURY RNA Isolation Kit™ (Exiqon, Copenhagen, Denmark) and cDNA was synthesized with the Universal cDNA Synthesis Kit™ (Exiqon, Copenhagen, Denmark). Real time PCR was performed by using SYBR® Green Master Mix Kit™ (Exiqon, Copenhagen, Denmark) and specific miR-92a primers (all consumables in this section were from Exiqon, Copenhagen, Denmark). Synthetic RNA spike-in templates and their primers (Exiqon, Denmark) were used for RT-PCR internal control. The ABI Step One Plus (ABI, USA) instrument was used for real time PCR experiments and the ΔΔCt method for data calculation.

### Cells Viability Assay

The viability of cells were assessed by the MTT (3-[4, 5 dimethylthiazol-2-yl]-2, 5-diphenyl tetrazolium bromide) assay, which is based on reduction of MTT by the mitochondrial dehydrogenase of intact cells to purple formazan products. The conversion is directly related to the number of living cells. The MTT assay was performed at the time intervals of 48, and 72 h post transfection. Two hundred microliters of MTT (Sigma-Aldrich, USA) at the concentration of 50 mg/mL was added to 5x105 HL-60 cells suspended in 2 mL of RPMI 1640 medium and incubated for 4 h at 37 °C in the dark. Two hundred microliters of dimethyl sulfoxide (DMSO; Sigma-Aldrich, USA) was added to each well and was shaken until dissolution of crystals. Blank samples were prepared using exactly the same procedure with an exception of cell incorporation. Absorbance was measured by using a spectrophotometer (PG Instrument T80, England) at 570 nm. Reading was converted to the percentage of the controls.

### Statistical Analysis

All the experiments were carried out in triplicate. The results were calculated by using the SPSS (version 16) software. The ANOVA test was used to assess differences the between the groups. Data were presented as mean±SD. Statistical significance was defined as p<0.05.

## RESULTS

### miRCURY LNA microRNA Inhibitor™ Strongly Inhibits miR-92a

**Figure 1:**
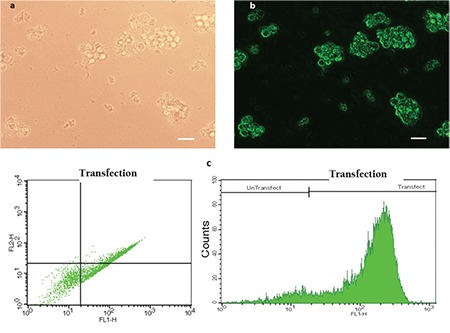
HL-60 cells have been transfected with 6-FAM™ fluorescein-conjugated LNA oligonucleotides and then to assess transfection efficiency, they have been observed by a fluorescent microscope and analyzed by flow cytometry. Phase contrast (a) and fluorescent (b) images of the same field of HL-60 cells show that a majority of the cells were transfected. Representative FSC-SCS and FL1-Count flow cytometry graphs are shown in (c). Scale bars: 50 µm.

For inhibition of miR-92a, the miRCURY LNA microRNA Inhibitor™ was transfected to HL-60 cells with the X-tremeGENE siRNA Transfection Reagent. On the basis of the initial optimization experiments, transfection was performed with 50 pM of LNA-anti-miR and 5 µL of the transfection reagent. As the transfected oligonucleotides were fluorochrome-conjugated, transfection efficiencies were assessed by fluorescence microscopy and flow cytometry. Carefully optimized protocol produced transfection efficiency of 90% [Figure 1].

**Figure 2:**
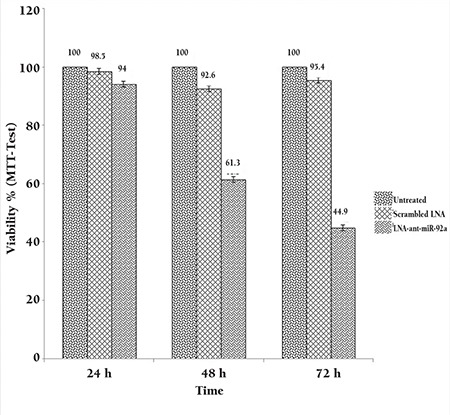
Assessment of the miR-92a level by real time PCR 24, 48, and 72 h after transfection. The ΔΔCt method was used for data analysis, and the untreated group was considered as a reference for each time point. Data were mean±SD of three independent experiments.

Expression of miR-92a was evaluated by reverse transcriptase microRNA real time PCR in HL-60 cells transfected with the miRCURY LNA microRNA Inhibitor™ (LNA-anti-miR group), the microRNA inhibitor scrambled oligonucleotides (scrambled LNA group), and untreated HL-60 cells (untreated groups), at 24, 48, and 72 h post-transfection. Although, miR-92a expression was a little lower in the scrambled LNA-transfected cells compared to the untreated cells, the differences were not statistically significant. However, in all three time points, the expression of miR-92a was considerably lower in the LNA-anti-miR group compared to the control groups (p<0.025). The expression of miR-92a was at the lowest level 24 h after transfection and gradually increased in the next two time points [Figure 2].

### Inhibition of miR-92a Decreased Viable HL-60 Cells

**Figure 3:**
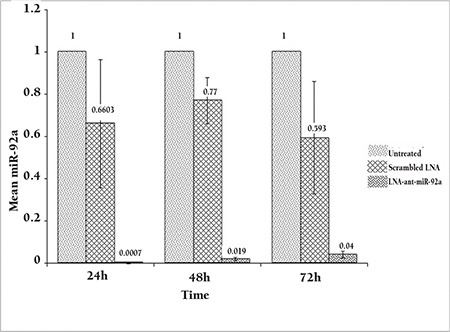
Assessment of cell viability by the MTT assay performed 24, 48, and 72 h after transfection. The viability of the untreated cells in each time point was considered as 100% and the viability of other groups is presented as the percentage of the untreated cells in the same time point. Data were mean±SD of three independent experiments.

To assess the effect of miR-92a inhibition on cell viability, the MTT assay was performed 24, 48, and 72 h after transfection. Cell viability was lower in the scrambled-LNA group compared to the untreated cells at 48 and 72 h after transfection (p<0.05). Cell viability of HL-60 cells was considerably decreased LNA-anti-miR post-transfection. The effect of LNA-anti-miR transfection on the cell viability gradually increased over time as the viability of the LNA-anti-miR transfected cell was less than 50% of the untreated cells at 72 h post-transfection [Figure 3]. The difference of the cell viability between the LNA-anti-miR and both control groups (untreated and scrambled treated) was statistically significant (p<0.024) at all three time points [Figure 3].

## DISCUSSION

AML, a prevalent malignancy of myeloid cells, is associated with a high rate of morbidity and mortality [16,15]. In spite of recent progresses, the therapeutic options for this disorder are still suboptimal and, therefore, novel therapeutic options are urgently needed [16,24,15]. MiRNAs are known to function as either oncogenes (oncomiRs) or tumor suppressors [25]. Reversal of the deregulated miRNAs normalized their cellular levels and hence provides blockage of the pathological pathways resulted from miRNAs ill expression, which is an alternative novel therapeutic option [21].

In this study, we have used LNA-anti-miR to the inhibition of miR-92a in APL (AML-M3) cell line. Real time PCR data confirmed that this miRNA was almost entirely down-regulated after LNA-anti-miR transfection. As well, the MTT assay showed that inhibition of miR92a is associated with decreased cell viability after 24, 48, and 72 h. Although, probably due to the transfection reagent toxicity, cell viability was minimally decreased in the scrambled LNA transfected cells compared to the normal controls (untreated cells) but this difference was not statistically significant.

MiR-92a is traced to be involved in various leukemia, including AML and ALL, hepatocellular carcinoma (HCC), and several other cancers [26]. It is suggested that miR-92a target’s estrogen receptor (ERβ1) mRNA hence down regulating its expression in breast cancer [27]. In AML, miR-92a overexpression was observed, as well as its inverse relationship with p63 protein expression in murine CD32myeloid cells [22,21].

There are a number of studies on oncomiRs as an approach to cancer treatment by employing an appropriate inhibitory molecule to down-regulate specific cellular miRNAs [28,29,30]. These studies are rapidly expanding into advanced phases expecting to generate a new path in challenges like chemo resistant cases or circumstances needing augmentation of weak response to therapies [25]. One of the technologies employed in oncomiRs inhibition is the LNA technology [31]. In this study, we used the same technology to suppress already up-regulated miR-92a in an APL cell line. Prevention of cellular proliferation subsequent to the LNA transfection is indicative of successful inhibition of miR92a in the cell line under study. Based on the fact that cytogenetic abnormality of APL is really complex and needs careful karyotype examination for patient’s stratification and design of the therapeutic plan, blockage of APL proliferation by inhibition of just one miRNA is a big achievement. Manni et al. reported in a mouse acute myeloid leukemia cell line that miR-92 inhibits p63 expression and that this protein has an important role in cell proliferation [22]. Perhaps in human APL, p63 expression is inhibited by miR-92a and induces the cell proliferation. Chemotherapy is a routine approach for AML treatment [15,16], However, in many cases, chemo resistance does not allow effective treatment and cell proliferation would not relief, as it is frequently exemplified by resistance to all-trans retinoic acid (ATRA) [24]. On the other hand, there are some instances that oncomiR inhibition acts to sensitize the cells to chemotherapy agents, making this combination therapy more effective than any one of the strategies used alone [32,33].

As a conclusion, taken together, our data suggest that inhibition of miR-92a with LNA-anti-miR may provide an alternative approach for the treatment of APL. It can be used alone or in combination with current therapies to reduce the existing limitations in the treatment of this malignancy. We realize that further in vivo studies are definitely required to assess the feasibility of this strategy. However, efficient in vivo delivery of anti-miR oligonucleotides remains an obstacle to be tackled before we can move to clinical trials.

## CONFLICT OF INTEREST STATEMENT

The authors of this paper have no conflicts of interest, including specific financial interests, relationships, and/ or affiliations relevant to the subject matter or materials included.
